# Hydrogen Sulfide and Carnosine: Modulation of Oxidative Stress and Inflammation in Kidney and Brain Axis

**DOI:** 10.3390/antiox9121303

**Published:** 2020-12-18

**Authors:** Vittorio Calabrese, Maria Scuto, Angela Trovato Salinaro, Giuseppe Dionisio, Sergio Modafferi, Maria Laura Ontario, Valentina Greco, Sebastiano Sciuto, Claus Peter Schmitt, Edward J. Calabrese, Verena Peters

**Affiliations:** 1 Department of Biomedical and Biotechnological Sciences, University of Catania, 95125 Catania, Italy; mary-amir@hotmail.it (M.S.); sergio.modafferi@gmail.com (S.M.); Marialaura.ontario@ontariosrl.it (M.L.O.); vgreco@unict.it (V.G.); ssciuto@unict.it (S.S.); 2 Department of Molecular Biology and Genetics, Research Center Flakkebjerg, Aarhus University, Forsøgsvej 1, 4200 Slagelse, Denmark; giuseppe.dionisio@mbg.au.dk; 3 Centre for Pediatric and Adolescent Medicine, University of Heidelberg, 69120 Heidelberg, Germany; clauspeter.schmitt@med.uni-heidelberg.de (C.P.S.); Verena.Peters@med.uni-heidelberg.de (V.P.); 4 Department of Environmental Health Sciences, Morrill I, N344, University of Massachusetts, Amherst, MA 01003, USA; edwardc@schoolph.umass.edu

**Keywords:** carnosine, hydrogen sulfide, inflammation, oxidative stress, vitagenes, kidney–brain axis

## Abstract

Emerging evidence indicates that the dysregulation of cellular redox homeostasis and chronic inflammatory processes are implicated in the pathogenesis of kidney and brain disorders. In this light, endogenous dipeptide carnosine (β-alanyl-L-histidine) and hydrogen sulfide (H_2_S) exert cytoprotective actions through the modulation of redox-dependent resilience pathways during oxidative stress and inflammation. Several recent studies have elucidated a functional crosstalk occurring between kidney and the brain. The pathophysiological link of this crosstalk is represented by oxidative stress and inflammatory processes which contribute to the high prevalence of neuropsychiatric disorders, cognitive impairment, and dementia during the natural history of chronic kidney disease. Herein, we provide an overview of the main pathophysiological mechanisms related to high levels of pro-inflammatory cytokines, including interleukin-1β (IL-1β), tumor necrosis factor-α (TNF-α), interleukin-6 (IL-6), and neurotoxins, which play a critical role in the kidney–brain crosstalk. The present paper also explores the respective role of H_2_S and carnosine in the modulation of oxidative stress and inflammation in the kidney–brain axis. It suggests that these activities are likely mediated, at least in part, via hormetic processes, involving Nrf2 (Nuclear factor-like 2), Hsp 70 (heat shock protein 70), SIRT-1 (Sirtuin-1), Trx (Thioredoxin), and the glutathione system. Metabolic interactions at the kidney and brain axis level operate in controlling and reducing oxidant-induced inflammatory damage and therefore, can be a promising potential therapeutic target to reduce the severity of renal and brain injuries in humans.

## 1. Introduction

The dysregulation of cellular redox homeostasis and chronic inflammatory processes represent interdependent factors implicated in the pathogenesis of multiple diseases, including atherosclerosis and cardiovascular diseases, neurodegenerative diseases, chronic kidney disease, diabetes, cancer, and aging [1,2]. Both inflammation and oxidative stress are orchestrated to accentuate each other and to induce progressive damage. Thus, their complex interrelations should be considered when evaluating novel therapies [2]. In this regard, many small molecules able to modulate endogenous cellular defense mechanisms and biochemical pathways of cytoprotection have demonstrated a major role for cellular protection in neurodegenerative disorders such as Alzheimer’s Disease (AD), Parkinson’s Disease (PD), Multiple Sclerosis (MS), and chronic inflammatory diseases such as Type 2 Diabetes mellitus (T2DM) [1]. The biological relevance of carnosine and its derivates and their metabolism is only partially understood but there is evidence that a group of histidine-containing dipeptides (HDPs) have a protective function in disease progression. Carnosine inhibits glycation [3], functions as a carbonyl scavenger [4,5,6], acts as an ion-chelating agent, especially for copper(II) and zinc(II) [7], and as an ACE inhibitor [8,9]. Several studies demonstrated a multifunctional antioxidant activity of carnosine [10,11,12,13], anserine, and homocarnosine [14,15]. Moreover, it is suggested that these activities are likely mediated, at least in part, via hormetic processes, involving Nrf2, Hsp70, Sirt-1, Trx, and the glutathione system [16]. The evidence to support this general perspective is strongly suggestive but limited in specificity. For example, Nrf2 activation can follow a hormetic biphasic dose response in various cell types [17,18]. There is also a substantial number of papers demonstrating the occurrence of H_2_S-induced hormetic dose responses in a broad variety of cell types such as cardiac [19], bone marrow stem cells [20], mammary epithelia cells [21], endothelial cells [22], lymphocytes [23], hepatocytes [24] as well as the brain [25] and neuro-stem cells [26]. However, research is needed to further explore whether and to what extent kidney–brain crosstalk occurs, its underlying mechanisms, whether effective treatment could be formulated, and how these factors may be related to hormetic processes. Despite these limitations, there is substantial documentation in the experimental literature that low doses of X-rays prevent the occurrence of diabetes-induced renal toxicity via the activation of the Nrf2 pathway. On the other hand, when blocking this activation, the protection disappears [27]. In a parallel manner, low dose X-ray treatment induces the development of a generalized anti-inflammatory phenotype that reduces arthritic inflammation in humans for prolonged periods [28,29]. Calabrese et al. (2019) [30] report that low dose X-ray treatments reduced inflammatory processes in numerous organs including the brain. Since Nrf2 activation is commonly induced via X-rays to affect these protective processes, it is likely that physical and/or chemical activators of Nrf2, such as H_2_S, may have clinical application within the context of the kidney–brain axis, as therapeutic modulation of these protective pathways can be mutually beneficial. The brain and the kidney interact strongly, and patients with neuropsychiatric disorders have a higher frequency of renal diseases. The crosstalk between the two organs may be caused by inflammatory processes via cytokine/chemokine release, oxidative stress via production of ROS, and factors related to the renin–angiotensin system [31]. 

Inflammation and oxidative stress are tightly linked and are emerging as key factors in several chronic diseases. Understanding the interrelation between oxidative stress and inflammation and the antioxidative and anti-inflammatory protective effects of carnosine and H_2_S in kidney and brain pathophysiology may lead to new therapeutic interventions and are now discussed.

## 2. Carnosine Signaling in Kidney and Brain

Carnosine (ß-alanyl-L-histidine), anserine (ß-alanyl-N^π^-methyl-histidine), homocarnosine (γ-aminobutyryl acid-L-histidine), and ophidine/balenine (ß-alanyl-N^τ^-methyl-histidine) belong to the HDPs. These dipeptides are found in humans, mammals, fish, and amphibia and their ratio and concentrations vary widely. In mammals and humans, carnosine seems to be the most important dipeptide, with the highest concentrations in the muscle (up to 20 mM), mostly together with either anserine or ophidine in different ratios [32]. Their abundant presence in muscles has been linked to their ability to counteract the massive production of lactic acid occurring during intense muscle activity, thus preventing a decrease in cytosolic pH [33]. Carnosine is also present in the kidney of mice [34,35], retina [36], liver [37], and spleen [38]. The highest carnosine concentrations in the brain were found in the olfactory system and carnosine levels are comparable to those usually found in skeletal muscle. However, homocarnosine is the most prevalent dipeptide in the mammalian brain [39] and this has been attributed to the bioavailability of GABA (γ-aminobutyric acid), the non-proteinogenic precursor of homocarnosine, exclusively present in the brain areas [40].

### 2.1. Carnosine in Cell Lines

Evidence from studies on cerebral cell cultures showed that carnosine is produced by glial cells [39]. The extensive distribution of glial cells in the brain and spinal cord suggest a broad spectrum of function and a diffuse presence of carnosine and its related dipeptides in the central nervous systems [40]. Carnosine is metabolized by two carnosinases, members of the M20 family of metalloproteases. The two isoforms carnosinase 1 (CN1) and carnosinase 2 (CN2) are structurally similar, but have enough varying properties. CN1 has a narrow substrate spectrum for histidine-containing dipeptides, such as carnosine, anserine, and homocarnosine. Conversely, the cytosolic isoform CN2 degrades a great number of dipeptides, but not homocarnosine [41]. The human kidney possesses an intrinsic carnosine metabolism. Distribution of carnosinase varies within the nephron regulating renal physiology. In the distal tubules, where a continuous low pH is required, the high levels of CN1 assure the removal of carnosine, which has high pH buffering capacity. Furthermore, the high levels of CN1 mRNA, proteins, and enzyme activities measured in podocytes, and the consistently low levels found in endothelial cells, suggest a cell-specific role of carnosine metabolism [42]. Carnosine prevented protein oxidation induced by glucose oxidase. In addition, carnosine proved to reduce both poly(ADP-ribose) polymerase-1 (PARP-1) and poly(ADP-ribose) polymerase-2 (PARP-2) activation induced by oxidative stress [43]. In contrast, carnosine is only transported slowly into renal cells [34], it can penetrate neurons [44], and several neuroprotective effects have been reported for carnosine [45]. Carnosine has the ability to protect neuronal cells against ischemic injury and oxidative stress [46]. In astrocytes, pre-treatment with carnosine reduced the overexpression of inducible isoform nitric oxide synthases (iNOS) caused by nitrosative stress. The direct link with nitric oxide is one of the possible mechanisms by which carnosine is able to neutralize the pathological effects of nitrosative stress [47]. Preston et al. [48] first reported a protective effect of carnosine on the cellular damage induced by amyloid-beta (Aβ) toxicity in the rat brain.

### 2.2. Carnosine in Animal Models

In rodents, the effects of carnosine supplementation have been extensively studied, especially in diabetes [49] and in neurological functions [40]. In diabetic mice and rats, renal carnosine metabolism is altered and exogenous carnosine intake exerts a range of nephroprotective effects such as a reduction in proteinuria, renal vasculopathy, and podocyte loss [34,35]. Carnosine can mitigate nitrite-induced metabolic alterations and oxidative damage in Wistar rats by increasing plasma GSH levels and major antioxidant defense enzymes, such as superoxide dismutase (SOD), glutathione reductase, or glutathione peroxidase [50] and decreasing inflammatory molecules, such as TNF-α and CRP (C-reactive protein) levels [51]. Recently, a carnosinase-resistant carnosine derivate was shown to prevent the onset of diabetic nephropathy in diabetic (db/db) mice by promoting renal inflammation and injury [52]. In stimulated murine macrophages, carnosine decreased apparent NO formation and enabled the modulation of macrophage-mediated inflammation processes [53]. In mice with chronic methylglyoxal administration, the generated hepatic and plasma oxidative stress was suppressed by carnosine treatment [54]. In Zucker obese rats, the beneficial effects of carnosine seem to be mediated by disruption of the advanced lipoxidation/glycation end products–receptor for advanced glycation end products (ALEs/AGEs-RAGE)–pro-inflammatory axis [55]. Intraperitoneal injection of carnosine protected against white matter damage caused by chronic cerebral ischemia in mice, likely by reducing oligodendroglial cell loss [56]. Carnosine inhibited microglia activation and cortical neuron apoptosis in a rat model of experimental subarachnoid hemorrhage [57]. In salsolinol-induced neurotoxicity in rats, cytotoxicity was reverted by treatment with carnosine. The latter has normalized the levels of malonaldehyde, glutathione, superoxide dismutase, and catalase [58]. In STZ-induced diabetic rats, carnosine treatment ameliorated learning and memory disturbances through modulation of the NF-κB/Nrf2/HO-1 (nuclear factor kappa-light-chain-enhancer of activated B cells/Nuclear factor-like 2/heme oxygenase-1) signaling cascade, with suppression of oxidative stress, neuroinflammation, astrogliosis, and enhancement of cholinergic function [59].

### 2.3. Carnosine in Clinical Settings

Increased concentrations of carnosine or homocarnosine due to carnosinemia caused by serum carnosinase deficiency seem to be clinically irrelevant under physiological conditions, but it has been reported that increased levels may be of therapeutic relevance, particularly in conditions exacerbated by oxidative stress. In humans, the half-life of carnosine in the human circulation is minutes only, even in subjects with low CN1 activity and protein content [60], but surprisingly, carnosine supplementation in humans showed beneficial effects. Besides treatment options in cancer, cataracts, and cachexia, several studies showed the beneficial effect of carnosine on glucose metabolism and neurological functions. Carnosine normalized glucose intolerance and reduced 2-h insulin levels after an oral glucose tolerance test (OGTT) in a subgroup of individuals with impaired glucose tolerance [61]. Recently, it was shown that carnosine lowered fasting glucose, serum levels of triglycerides, AGEs, and TNF-α without changing sRAGE, IL-6, and IL-1β levels in type 2 diabetes patients [62]. In pediatric patients with diabetic nephropathy, oral supplementation with L-Carnosine reduced oxidative stress, increased antioxidant levels and low malondialdehyde levels, and improved glycemic control (decreased HbA1c, insulin resistance, increased insulin secretion, and β-cell mass). It also improved renal function [63]. CN1 inhibitors or medications increasing internal synthesis are currently being developed [64]. In human plasma, all HDPs are present at very low concentrations, with the highest levels of homocarnosine, while the mean concentration of carnosine and ophidine/balenine was below the detection limit [65].

HDP can also improve neurological diseases. Topiramate has been described to increase brain GABA and homocarnosine levels that could contribute to enhance its potent antiepileptic action in patients with complex partial seizures. A randomized double-blind placebo-controlled study on 75 patients with schizophrenia has demonstrated that carnosine (2 g/day) adjunctive to basic therapy improves cognitive functions [66]. Carnosine plays a protective role in neurodegenerative disorders through several mechanisms. Many other studies confirmed these effects, suggesting that carnosine reverses the neurotoxicity induced by Aβ (1–42) by inhibiting glutamatergic activity [12]. In spite of its function as a molecular chaperone, carnosine has recently been proposed to inhibit Aβ aggregation by interfering with the propensity of the peptide to form backbone hydrogen bonds near residues with key roles in fibrillogenesis [67]. A pilot study with 52 patients diagnosed with moderate Alzheimer’s disease and treated with the acetylcholinesterase inhibitor donepezil reported that the group that received carnosine (along with an antioxidant cocktail) showed improvements in their Mini-Mental Status Exam II scores, while the group that continued to receive only donepezil treatment maintained similar scores [68]. Moreover, a recent double-blind, randomized clinical trial of 43 autistic patients has demonstrated that 500 mg of carnosine improved sleep duration and parasomnia subscales [69]. In addition, another recent randomized, double-blind, placebo-controlled trial has suggested that 250 mg of oral carnosine supplementation exerts protective effects against cognitive decline in APOE4 (+) mild cognitive impairment (MCI) patients [70]. In addition, a double-blind randomized controlled trial of 60 AD patients preserve verbal episodic memory, probably owing to inflammatory chemokine CCL24 suppression in the blood [71]. Carnosine has been also proposed as a potential drug for the treatment of Parkinson’s disease. It has been described to enhance the efficacy of the levodopa (L-DOPA)-based treatment of Parkinson’s disease and to inhibit the a-synuclein oligomerization induced by the Cu, Zn-SOD, and hydrogen peroxide system [72]. The oxidative and nitrosative stresses represent other characteristic aspects of neurodegeneration which could be regulated by carnosine. Interestingly, carnosine treatment induced a prominent reduction in intraneuronal Aβ in the hippocampus of transgenic mice, but failed to decrease phospho-tau immunoreactive levels and to restore long-term memory deficits. Thus, the reduction in Aβ by carnosine appears to be not sufficient to produce an appreciable cognitive improvement. Furthermore, lower plasma levels of carnosine have also been revealed in Alzheimer disease patients with respect to age-matched controls [73].

## 3. Hydrogen Sulfide Signaling

**Figure 1:**
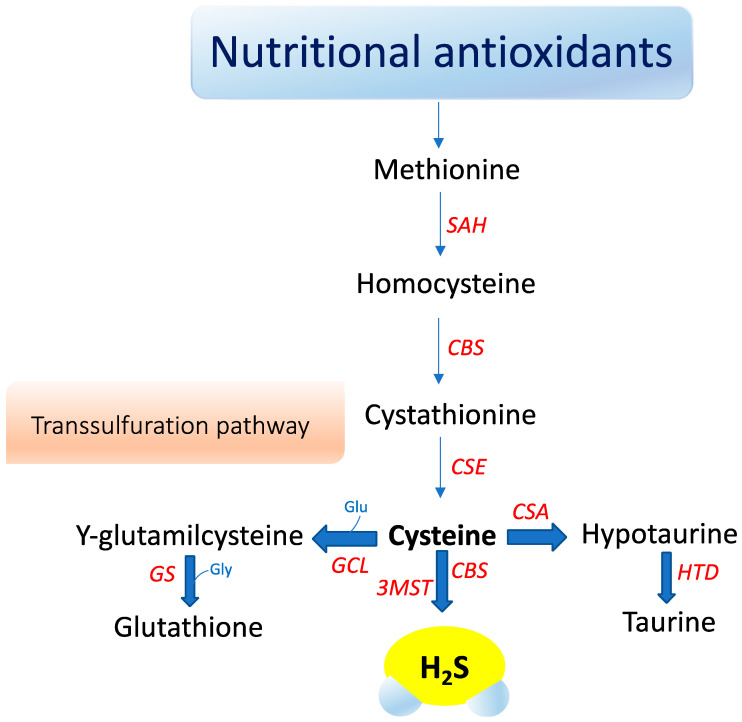
Nutraceutical support of intracellular H_2_S synthesis via cysteine metabolism. S-adenosyl homocysteine hydrolase (SAH), Cystathionine-β-synthase (CBS), 3-mercaptopyruvate sulfurtransferase (3-MST), Cystathionine gamma lyase (CSE), Cysteine sulfinate (CSA), Hypotaurine dehydrogenase (HTD), Glutamate cysteine ligase (GCL), Glutathione synthetase (GS), Glycine (Gly).

Hydrogen sulfide (H_2_S) is a small gaseous molecule with profound biological effects within living organisms. It exerts key roles in cytoprotection, inflammation, vascular function, neurological systems, mitochondrial function, energy metabolism, and ageing [74,75]. However, H_2_S was originally known for its deleterious effects on health and the environment. First, in 1700, Italian physician Bernardino Ramazzini [76] described a severe ocular irritation and inflammation in sewer workers, caused by an unspecified volatile acid. Later, the chemical composition of H_2_S was elucidated and its association with ocular adverse effects and intoxication in sewer workers was recognized. For over a century, studies focused on its major toxic effects—e.g., inhibition of cytochrome c oxidase, carbonic anhydrase, monoamine oxidase, and sodium/potassium-ATPase (NaC/KC ATPase) [77]. The image of H_2_S was revolutionized when Kimura, in 1996, revealed its role as an endogenous neuromodulator [78]. Recently, H_2_S was classified as the third gasotransmitter along with NO and carbon monoxide (CO). H_2_S is enzymatically released in our body and it has a highly regulated metabolism. It is freely permeable to membranes and exerts specific physiological functions in several systems that can be mimicked by H_2_S donors applied exogenously [79]. In mammals, hydrogen sulfide (H_2_S) is primarily produced by two cytosolic pyridoxal-5′-phosphate-dependent enzymes, cystathionine β-synthase (CBS) and cystathionine γ-lyase (CSE), which use the substrates homocysteine and L-cysteine (Figure 1).

A third enzyme, 3-mercaptopyruvate sulfurtransferase (3-MST), catalyzes H_2_S production mainly in mitochondria by the conversion of 3-mercaptopyruvate to pyruvate and H_2_S [80]. Recently, an additional biosynthetic pathway has been described for the production of hydrogen sulfide from D-cysteine involving 3-mercaptopyruvate sulfurtransferase and D-amino acid oxidase that operates predominantly in the cerebellum and the kidney, i.e., within the KB axis [81]. D-cysteine is mainly adsorbed with food and derives from L-cysteine racemization during food processing. This novel pathway is of particular interest since supplementation with D-cysteine showed to protect renal cortex cells and cerebellum cells (i.e., KB axis) more efficiently than L-cysteine [81]. Furthermore, it was reported that gut microbiota would be another source of H_2_S that might influence health and function [81]. The contribution of each of these enzymes to net H_2_S production is dictated by its presence and relative tissue concentration, which varies in a cell-specific manner [81,82,83]. CBS is the major H_2_S-producing enzyme in the brain, while CSE is significantly expressed in the mammalian cardiovascular system and respiratory system and it seems to be the main H_2_S-forming enzyme in the liver, kidney, and pancreas [84].

Oxidative stress seems to influence CSE and CBS in a different manner. Reactive oxygen species appear to induce CSE expression, whereas they clearly suppress the transcription of the human CBS gene [85]. Eventually, H_2_S is metabolized to sulfite in the mitochondria by thiosulfate reductase, and then, is oxidized to thiosulfate and sulfate by sulfite oxidase. The sulfates are excreted in the urine [80]. Interestingly, urinary sulfates have been used as markers of H_2_S plasma levels and increased urinary sulfate concentrations have been correlated with a decreased risk of renal events in type 2 diabetic patients with nephropathy [86]. However, urinary sulfate and thiosulfate are not specific markers for endogenous H_2_S formation, and can also be the products of exogenous H_2_S production by sulfate-reducing bacteria in the gut [86]. H_2_S acts independently of any specific transporters by mechanisms not fully understood. S-sulfhydration, a novel posttranslational modification, is emerging as a mechanism responsible for many biological effects mediated by H_2_S [75,87]. H_2_S sulfhydrates protein thiol groups by transferring its sulfhydryl group to the cysteine residue of targeted proteins. Furthermore, H_2_S is oxidized in biological systems to polysulfides, which are now increasingly recognized as effectors of the H_2_S signaling mechanism [88]. H_2_S S-sulfhydration of enzymes, transcription factors, and ion channels has been described accounting for several protective effects of H_2_S, ranging from response to inflammation to cytoprotection [82,89]. For instance, H_2_S attenuates inflammation through the S-sulfhydration of Nuclear Factor-kappa B (NF-κB) [90]. H_2_S increases the antioxidative properties of cells by sulfhydration of Kelch-like ECH-associated protein 1 (Keap1), leading to its dissociation from Nrf2, which translocates in the nucleus and binds to the antioxidant response element (ARE) promoting antioxidant gene transcription, such as GCLM, GCLC, and glutathione reductase [91]. S-sulfhydration may be involved in the augment of the life span of *Caenorhabditis elegans* induced by H_2_S through sirtuins [92]. H_2_S may also inhibit mitochondria ROS production through sulfhydration of p66Shc [91]. In this regard, gut microbiota has attracted considerable interest for its role in microbial-mediated ROS generation, which might influence many signaling and homeostatic processes. Notably, lactobacilli ROS-mediated signaling has been described to induce Nrf2, opening the prospect that probiotic bacteria may elicit beneficial effects on disease states that involve Nrf2, including diabetes and neurodegenerative diseases [93]. In particular, in the (nod-like receptor) NLR family, the nod-like receptor pyrin domain-containing 3 (NLRP3) inflammasome has been reported to play a pathogenic role in the initiation and progression of metabolic and neurodegenerative diseases [94,95]. Recently, in vitro and in vivo studies showed that H_2_S mitigated lipopolysaccharide (LPS)-induced sepsis against oxidative stress and inflammation damage mediated by the NADPH oxidase 4 (Nox4) pathway [96], inhibiting the vicious cycle of NLRP3 inflammasome and oxidative stress in human retinal pigment epithelial cells [97] and in hypertensive rats [98]. In addition, H_2_S mediated effects on neuroinflammation and Aβ_1-42_ production by suppressing the activation of STAT3 and cathepsin S [99]. H_2_S suppresses oxidative stress-induced mtROS production and NLRP3 inflammasome activation via S-sulfhydrating c-Jun at cysteine 269 in macrophages [100].

## 4. H_2_S as a Signaling Mediator in the Kidney–Brain Axis

Over the past few years, a crosstalk between kidney and brain emerged, as elucidated through the modulation of H_2_S for neuroprotection, particularly to unveil the pathophysiological mechanisms of oxidative and inflammatory stress. Consistent with this notion H_2_S, as a biological signaling mediator, is involved in various functions, including antioxidant, neuromodulatory, regulation of vascular tone, cytoprotective, anti-inflammatory, modulator of immune response, oxygen sensing, angiogenesis, mitochondrial bioenergetics, and blood–brain barrier permeability [101]. The kidney and brain are vital organs exposed to high-volume blood flow and thus, are highly susceptible to vascular damage [102]. Normal renal function regulates whole-body homeostasis, including neuronal homeostasis [103]. Therefore, it was well recognized that oxidative stress and inflammation are common outcomes strongly associated with both kidney and brain dysfunctions, leading to the development and progression of several diseases, including acute kidney disease (AKD), chronic kidney disease (CKD), neuropsychiatric disorders, and cognitive dementia [104,105]. Recent data have demonstrated that patients with CKD, especially at advanced stages, are highly susceptible to developing neuropsychiatric disorders (i.e., depression and anxiety) and cognitive impairment (i.e., Alzheimer and Parkinson diseases) [31]. In the kidney, H_2_S induces important diuretic, natriuretic, and kaliuretic effects by raising glomerular filtration rate and inhibiting tubular sodium re-absorption [106,107]. Notably, under hypoxic conditions, H_2_S functions as an oxygen sensor in the renal medulla that restores oxygen balance by enhancing medullary blood flow, decreasing energy requirements for tubular transport and directly suppressing mitochondrial respiration [107]. Since medullary hypoxia is a common feature of CKD, H_2_S deficiency can lead to progression of CKD by limiting this significant adaptive mechanism [108].

### 4.1. Anti-Inflammatory Role of H_2_S

Inflammation is a common feature in brain and kidney injuries, and it is quite reasonable to assume that inflammatory mediators may amplify the kidney–brain crosstalk. Particularly, the nucleotide-binding oligomerization domain NLRP3 inflammasome, a multiprotein complex, is involved in the pathogenesis of inflammation in chronic kidney and brain diseases [109,110]. NLRP3 induces the production of many proinflammatory cytokines and chemokines such as IL-1β, IL-6, TNF-α, transforming growth factor-β (TGF-β), and NF-κB, which are frequently associated with the pathogenesis of CKD [111]. These cytokines then initiate or amplify different downstream signaling pathways and drive proinflammatory factors [112], leading to cellular damage, such as autophagy dysfunction and ROS production [113]. In the kidney, generation and activation of the NLRP3 inflammasome have been reported to occur not only in immune cells like dendritic cells [114] and infiltrating macrophages [115], but also in other renal cells such as tubular epithelial cells [116] and podocytes [117]. Several human studies reported that the inflammatory response triggered by CKD may contribute toward increasing proinflammatory cytokines that can cross the blood–brain barrier (BBB) to reach the CNS and lead to neuroinflammation such as reported with neuropsychiatric and neurodegenerative disorders, including Alzheimer’s disease, Parkinson’s disease, multiple sclerosis, major depressive disorder, and schizophrenia. This reinforces the concept of kidney–brain inflammatory crosstalk [118,119,120]. Accordingly, these pro-inflammatory molecules operate in the brain to induce common symptoms of sickness, including loss of appetite, sleepiness, withdrawal from normal social activities, fever, aching joints, fatigue, embezzlement of cognition, malaise, inattention, and depression [121]. In this context, H_2_S possess powerful anti-inflammatory, antioxidant, and anti-apoptotic effects [122]. On the other hand, H_2_S depletion may contribute to the progression of CKD and cognitive disorders [123,124]. Moreover, accumulating evidence reported that H_2_S appears to play a crucial role in the modulation of the immune response by regulating posttranslational modification of the NF-kB pathway in vitro and in vivo [125,126]. In the brain, H_2_S protects neurons from apoptosis and degeneration by inducing anti-inflammatory effects and upregulating antioxidant enzymes [127]. Additionally, treatment of H_2_S in the APP/PS1 (human amyloid precursor protein and presenilin 1) mouse model of AD reduced cognitive impairment and mitigated oxidative stress [128]. Moreover, H_2_S mediated the process of altered blood pressure in response to changes in serum homocysteine levels by blocking the activation of extracellular signal-regulated kinase 1/2 (ERK1/2)-STAT3 signaling pathway [129], exerted antidepressant effects by the induction of the mTORC1-TrkB-AMPA receptor pathway [130,131] as well as prevented cisplatin-induced nephrotoxicity [132]. Furthermore, NaHS, an exogenous H_2_S donor, also inhibits macrophage pro-inflammatory cytokine production, as well as cyclooxygenase-2 and nitric oxide production, and decreases macrophage motility. In addition, recent in vivo studies reported that NaHS ameliorates CKD-mediated brain dysfunctions through interaction with NO signaling in the hippocampus [133], as well as improves apoptosis, inflammation, and autophagy, exerting nephroprotective effects against CKD in rats [134,135].

### 4.2. Antioxidant Role of H_2_S

During pathological conditions, there is a mutual upregulation between factors promoting inflammation and oxidative stress, which sustains CDK progression and neurological disorders [136]. Under physiological conditions, ROS provides beneficial effects, regulating cellular stress responses by redox-sensitive signaling pathways. Indeed, ROS control cellular growth, differentiation, and migration; regulate vascular tone and cellular adhesion, leading to the production of iNOS at the transcriptional and posttranscriptional level by redox-dependent NF-κB or mitogen-activated protein kinases (MAPKs); modulate immune response; and control angiogenesis and apoptosis [137]. When ROS generation is prolonged or excessive, harmful consequences are observed with peculiar changes in cellular proteins, lipids, and ribonucleic acids, leading to cell dysfunction or death. Numerous enzymes with antioxidant activity are involved in neutralizing ROS, including superoxide dismutase (SOD), γ-glutamyltransferase (GGT), glutathione (GSH), glutathione reductase (GSSG-Rd), glutathione peroxidase (GSH-Px), glutathione S-transferase (GST), catalase (CAT), and nuclear factor erythroid 2-related factor 2 (Nrf2) [138]. All these antioxidant pathways are differently expressed in various cells and organs including kidney and brain tissues [139,140]. Intriguingly, recent evidence highlighted the complex role of H_2_S as a direct antioxidant scavenger to inhibit ROS generation and the inflammatory process in the pathogenesis of kidney and brain injuries [141,142]. Additionally, the formation of free radicals (i.e., ROS and RNS) as signaling molecules to promote inflammation induces the activation of the transcription of NF-κB by upregulating the production of pro-inflammatory cytokines and chemokines, leading to the recruitment and activation of leukocytes and resident cells [143]. The “oxidative” linkage between CKD and its complications is achieved through several pathophysiological mechanisms, including mitochondrial dysfunction [144], uremic neurotoxin-induced endothelial nitric oxide synthase (eNOS) uncoupling [145], and increased nicotinamide adenine dinucleotide phosphate-oxidases (NADPH oxidases (NOX)) activity [146], myeloperoxidase (MPO) [147], but also antioxidant enzymes depletion due to dietary restriction, and/or decreased intestinal absorption [148]. In particular, preclinical and clinical studies confirmed beneficial effects by upregulation of the Nrf2-HO1 antioxidant pathway during acute and chronic kidney diseases [149,150]. In addition, activation of the glutathione peroxidase pathway reduced not only the oxidative stress but also the inflammatory process induced by the uremic neurotoxins on the endothelium during CKD [151]. On the other hand, glutathione peroxidase deficiency in kidney diseases contributed to the development of cardiac diseases risk due to an increase in ROS generation and inflammatory pathways [152]. Furthermore, CKD patients also display hypovitaminosis D [153] as well as hypoalbuminemia [154] and zinc deficiency [155].

Fascinatingly evidence demonstrated that NaHS suppresses the expression of the ROS-generating enzyme, NADPH oxidase (NOX) and its essential subunit, Rac-1, in cultured vascular smooth muscle cells [156]. Likewise, NaHS has been shown to lower NOX-4 expression and potentiates the antioxidant effects of apocyanin, N-acetyl-L-cysteine, catalase, superoxide dismutase, and GSH, in the brain endothelial cells [157]. In addition, H_2_S can scavenge and/or degrade lipid peroxides [158] and increases the production of GSH [148]. Moreover, intraperitoneal application of NaHS to pregnant rats protects fetal brains from ischemia-reperfusion injury by restoring the glutathione levels reduced by ischemia-reperfusion [156]. However, experimental evidence demonstrated the scavenging effect of H_2_S in the presence of intracellular glutathione concentration between 1 and 10 mM, and 1 and 100 μM of cysteine in neurons [159]. Thus, the suppression of oxidative stress by increased levels of glutathione should be more effective than ROS scavenging by H_2_S itself.

**Figure 2:**
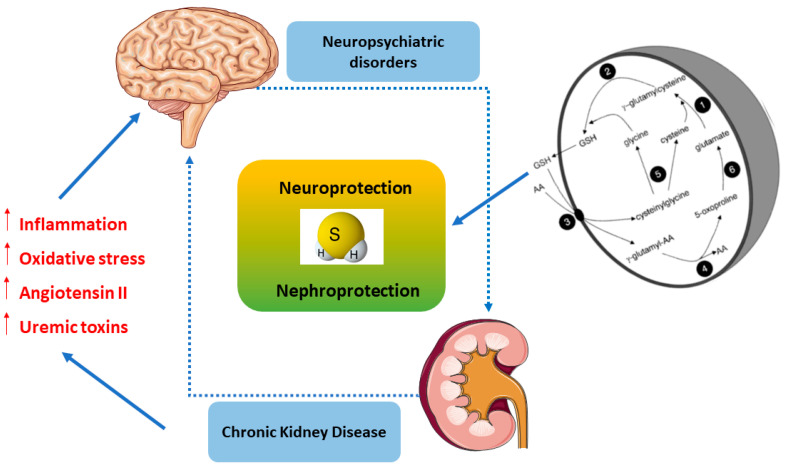
The kidney–brain crosstalk by the H_2_S signaling pathway. γ-glutamyl transpeptidase (γGGT) (3), γ-glutamyl cyclotransferase (4), dipeptidase (5), oxoprolinase (6), γ-glutamyl-cysteine synthase (1), and glutathione synthetase (2) operate in the Meister cycle to generate glutathione (GSH) and internalize amino acids (AA).

GSH is a ubiquitous thiol tripeptide composed of cysteine, glycine, and glutamate, existing often as a reduced form, and it is synthesized from cysteine. Moreover, H_2_S also facilitates the transport of cysteine into cells. Glutathione is produced by two enzymes, glutamate cysteine ligase (GCL) (γ-glutamylcysteine synthase (γ-GCS)), which is a rate-limiting enzyme in the production of γ-glutamyl cysteine from glutamate and cysteine, and glutathione synthetase (GS), which produces glutathione by adding glycine to γ-glutamyl cysteine. GSH reduces disulfide bonds formed within cytoplasmic proteins to cysteines by serving as an electron donor. In this process, GSH is converted to its oxidized form, glutathione disulfide (GSSG). Intracellular cysteine plays an important role in cellular homeostasis as a precursor for protein synthesis, and for the production of GSH, hydrogen sulfide (H_2_S), and taurine. Cysteine exists as two unstable redox forms in the body: the oxidized form cystine and the reduced form cysteine. The extracellular cystine form is carried into cells through the cystine/glutamate antiporter system, after which cysteine is reduced and ready for GSH synthesis. The release of H_2_S into the extracellular space promotes the reduction of cystine to cysteine, increasing the amount of cysteine available as a substrate for GSH synthesis, and improving cystine transport [156,160,161]. Another significant mechanism for the effect of H_2_S on GSH may be by enhancing glutamate uptake [162]. Interestingly, kidney and brain tissues contain significant amounts of γ-glutamyl transpeptidase and γ-glutamyl cyclotransferase and nevertheless, maintain an appreciable concentration of glutathione. This suggested to us that these organs might also possess the enzymatic equipment needed for the synthesis of glutathione. Many in vivo studies have demonstrated that the kidney–brain axis contains a high concentration of both γ-glutamylcysteine synthetase and glutathione synthetase [163,164]. The presence of these enzymes in kidney and brain enables a series of catalytic events involving the synthesis and degradation of glutathione, and the coupled uptake and release of free amino acids from γ-glutamyl linkage. These reactions are steps in a cyclical process referred to as the “γ-glutamylcycle” (Figure 2).

A great number of accumulated data indicate that oxidative stress and inflammation are major contributing factors of renin–angiotensin system (RAS) imbalance both in peripheral and brain tissues [165,166]. The potential homeostatic role between kidney–brain crosstalk by RAS to regulate sodium/water balance and maintain normal blood pressure has been purported [167]. The first end-product of this system related to a biological activity is the octapeptide angiotensin (ANG) II, synthesized by the angiotensin converting enzyme (ACE). This pathway may also be involved in neurocognitive performance decline [168]. The role of RAS peptides in the interaction between the kidney and brain is supported by studies showing that treatment with ACE inhibitors and type I receptor (AT1) blockers, besides exerting renoprotection, also have beneficial actions in neurodegenerative disorders [169]. Dysfunctional RAS activation induces angiotensin II release, increasing systemic vascular resistance [170]. It has been established that treatment with captopril reduces oxidative stress and protects dopaminergic neurons in a 6-hydroxydopamine rat model of Parkinson’s disease [171]. Analogue results were obtained with the administration of AT1 receptor antagonists in patients and in experimental models of Alzheimer’s disease, Parkinson’s disease, stroke, traumatic brain injury, and spinal cord injury [172]. Surprisingly, exogeneous H_2_S administration regulates renin release by downregulating the intracellular cAMP level in several cell types [173] and decreases protein expression of AT1R [174], resulting in renoprotection from kidney diseases. Moreover, H_2_S treatment inhibits the upregulation of renin level in renovascular hypertensive rats and reduces the intracellular cAMP level in primary cultures of renin-rich kidney cells [175]. In addition, H_2_S blocks forskolin-induced renin degranulation in mast cells by lowering the intracellular cAMP level, thus protecting against isoproterenol (ISO)-induced heart failure [176]. Likewise, H_2_S therapy protects multiple organs including the heart, kidney, and blood vessels and improves exercise capacity, coupled with inactivation of RAS in a murine model of transverse aortic constriction-induced heart failure [177]. Recent in vivo studies suggested that treatment with NaHS, increases renal H_2_S concentrations, restores NO bioavailability, and blocks RAS in the kidney, thus promoting vasodilatation to prevent the development of hypertension in rats [178]. In addition, H_2_S mitigates the development of diabetic nephropathy through suppressing RAS activity in diabetic rats [179].

As a rationale for neuropsychiatric disorders secondary to kidney damage, known as the “vascular theory”, kidney and brain hemodynamic analogies occur, both being low resistance end organs exposed to high-volume blood flow and, consequently, more vulnerable to vascular damage [180]. Accordingly, magnetic resonance imaging (MRI) studies have shown high occurrence of silent brain infarction in patients with CKD, supporting the vascular theory [181]. Consistent with this, approximately 50% of CKD patients present ischemic white matter lesions in the MRI compared with 10% of the general population [182]. The loss of a direct correlation between known vascular risk factors, like diabetes and hypertension with CKD-related cognitive decline [183], the onset of neuropsychiatric comorbidities in pediatric patients with CKD preceding the vascular damage [184], as well as inconsistent findings regarding antihypertensive drugs’ beneficial effects in cognition [185], may indicate that other mechanisms underlie CKD-associated brain dysfunctions. Cerebrovascular injuries in CKD have also been associated with the retention of uremic toxins along with electrolyte imbalance, which ultimately leads to neuropsychiatric diseases, especially cognitive impairment and dementia [186]. Importantly, high neurotoxin levels (up to 10-fold higher in CKD patients than in controls) of guanidino compounds were present in brain regions that play a determinant role in cognition, such as the thalamus, the mammillary bodies, and the cerebral cortex [187]. A recent study reported several uremic toxins that potentially mediate the interactions between kidney and brain, which in turn, may influence brain homeostasis. Convincingly, uric acid, indoxyl sulfate, p-cresyl sulfate, IL-1β, IL-6, TNF, and parathyroid hormone have a strong impact on cognition in uremic conditions [188]. Interestingly, uremic neurotoxic effects seem to be also mediated by guanidino compounds, including creatinine, guanidine, guanidinosuccinic acid, and methylguanidine. Indeed, some studies reported that guanidine compounds affect the CNS by inhibition of GABA_(A)_ receptors and concomitant activation of N-methyl-d-aspartate (NMDA) receptors [189,190]. Moreover, other studies showed that uremic mice decrease central dopamine turnover in the striatum, mesencephalon, and hypothalamus, which was correlated with the impairment of motor activity [191].

In this scenario, H_2_S plays a beneficial role by inhibiting uremic toxins release in vitro and in vivo. During uremia, CSE activity and also H_2_S are significantly decreased in blood mononuclear cells from uremic patients on hemodialysis [192]. On the contrary, its metabolic-related compounds such as cystathionine, homolanthionine, and lanthionine are significantly raised [193]. CSE inhibition could be due to a uremic toxin, lanthionine, which is able to decrease H_2_S production in hepatoma cells. Recent in vitro studies suggested that slow-releasing H_2_S donors, such as diallyl disulfide (DADS) and diallyl trisulfide (DATS) enhance levels of alkaline phosphatase, osteopontin, osteocalcin, and collagen type I that are downregulated in human mesenchymal stem cells derived from serum of uremic patients in hemodialysis [194]. Although the potential role of uremic toxins in mediating the kidney–brain crosstalk has become clearer over the past years, the role exerted by H_2_S on these neurotoxic compounds and how it may directly or indirectly influence these compounds and restore the CNS function remain still elusive.

Moreover, hyperhomocysteinemia is a clinical hallmark in patients with CKD or AKI, in the latter setting often caused by ischemia-reperfusion. It causes arteriolar constriction, arterial stiffness, and endothelial damage [195]. H_2_S treatment in rodents attenuates renovascular damage by reducing hyperhomocysteinemia [196]. The pathophysiology of chronic kidney disease and neuropsychiatric disorders has been commonly associated with mechanisms related to the decreased availability of brain-derived neurotropic factor (BDNF) for renal and neuronal failure [197]. BDNF exerts traditional antidepressant actions, and its deletion in the hippocampus weakens antidepressant behavioral responses [198]. Interestingly, epidemiological and experimental studies point to a potential role of the endogenous NOS inhibitor asymmetric dimethylarginine (ADMA) and BDNF in neuropsychiatric disorders, in particularly in depression [199]. BDNF regulates the growth and maintenance of the neuronal system as well as neuronal plasticity, like long-term potentiation of learning [200], which has been shown to be reduced in major depressive disorders [201]. Several preclinical and clinical data reported that an increase in ADMA infusion alone causes a marked reduction in serum BDNF levels leading to behavioral changes and depression of CKD in 11 hemodialyzed patients as well as in nephrectomized rats. Thus, ADMA is considered a uremic toxin that acts as an endogenous inhibitor of NO [197]. Recently, it has been reported that BDNF and tropomyosin receptor kinase B (TrkB) are expressed in podocytes and in the zebrafish model, being essential for actin polymerization and cell survival [202]. Moreover, a recent study demonstrated that *BDNF* is required for glomerular development, morphology, and function, and the expression of *BDNF* and *KIM-1* is highly correlated in urine cells of CKD patients. Therefore, *BDNF* mRNA in urine cells could serve as a potential prognostic biomarker for CKD [203]. Some studies demonstrated that H_2_S reversed the decrease in TrKB receptors against chronic unpredictable mild stress (CUMS)-induced hippocampal oxidative stress, demonstrating the critical role of neurotrophic signaling in the antidepressant effects mediated by H_2_S [204]. These outcomes are consistent with premises that H_2_S exerted neuroprotective effects against formaldehyde-induced toxicity in PC12 cells [205] as well as against homocysteine-induced ER stress and neuronal apoptosis in the hippocampus of rat via the BDNF–TrKB pathway [206]. Taken together, the data indicate that the interactions between kidney and brain are complex and multifaceted, thus justifying the significant neuropsychiatric comorbidity observed in patients with CKD. Despite much research efforts, to date, kidney–brain crosstalk is still an area with excitingly few publications, especially as regards the molecular mechanisms underlying CDK and brain damage. Consistent with this, H_2_S signaling could represent a novel direct link between kidney and brain diseases, conferring protection, and limiting neuropsychiatric disorder occurrence in CKD patients.

## 5. H_2_S and Diabetes

H_2_S is abundant in kidney and is generated mainly by CBS and CSE. In the glomeruli, CSE is the main H_2_S-producing enzyme expressed by endothelial cells, mesangial cells, and podocytes [207], while both CBS and CSE have been reported to be expressed on renal proximal tubules [78]. H_2_S is important in kidney for its role in homocysteine metabolism, regulation of GFR and of urinary sodium and potassium excretion [76]. In a murine model of renovascular hypertension, NaHS, a donor of H_2_S, suppressed the upregulation of renin mRNA by downregulating cAMP levels [208]. Emerging evidence indicates an active role of H_2_S in diabetes and in diabetic nephropathy (DN). CSE expression is upregulated in liver and pancreas in streptozotocin-induced diabetic rats, a model for type-1 diabetes. Insulin treatment reduced CSE expression [81]. CSE is also overexpressed in pancreatic β-cells in Zucker diabetic fatty rats, a model for type-2 diabetes [209]. On the other hand, renal expression of CBS and CSE enzymes which synthetize H_2_S, has been reported downregulated both in patients and in animal models of diabetes [76]. Hyperglycemia impairs cell redox homeostasis increasing ROS generation, reducing the activities of endogenous antioxidants such as GSH and SOD and downregulating Nrf-2, which control the expression of protective enzymes [207]. In addition, hyperglycemia-induced oxidative stress reduced CBS and CSE expression by upregulating MMP-9. These effects have been reported in cultured mesangial cells [210] and in diabetic rats [211] treated with high glucose and were reversed by treatment with NaHS [78]. H_2_S have been also reported to attenuate renovascular remodeling in DN through its regulatory action on MPP-9 and NADPH oxidase 4 (NOX4) [212,213]. In renal epithelial cells, NaHS reversed the inactivation of AMPK, responsible for the cascade that leads to hyperactivation of mTOR and subsequent matrix deposition [214]. Later, it was demonstrated that H_2_S inhibits NOX4 expression and matrix deposition by activation of iNOS and NO production [94]. Interestingly NO in turn is able to induce CSE and H_2_S production, suggesting a crosstalk interaction between the two gasotransmitters [215]. H_2_S have demonstrated an antifibrotic effect in renal tubular epithelial cells by attenuating the TGF-beta1-induced epithelial-to-mesenchymal transition (EMT) through both ERK-dependent and β-catenin-dependent pathways [216]. In addition, H_2_S accelerates wound healing in diabetic rats [217]. H_2_S levels and H_2_S-producing enzymes were found decreased in plasma, urine, and kidney of aging mice. Chronic H_2_S donor (NaHS) treatment could attenuate oxidative stress levels and renal tubular interstitial collagen deposition and restores H_2_S production in aging kidney. These protective effects may refer to Nrf2 activation and transcription of antioxidant proteins, including HO-1, SIRT1, SOD1, and SOD2, which are upregulated in the ageing kidney after NaHS treatment [218].

## 6. H_2_S and Brain

In the brain, CBS is the main H_2_S-producing enzyme, present in both neurons and astrocytes [219], and it was expressed mainly in the hippocampus and cerebellum when compared with the cerebral cortex and brain stem [220]. Furthermore, H_2_S production in astrocytes is approximately 10-fold higher than in cultured microglial and neuronal cells, suggesting that astrocytes are the main brain cells producing H_2_S [1]. H_2_S and sulfhydration play significant roles in the optimal functioning of the nervous system. For instance, H_2_S takes part in the regulation of intracellular Ca^2+^ in neurons and microglia, maintains pH homeostasis [76], and induces hippocampal long-term potentiation in active synapsis by activating N-methyl-D-aspartate (NMDA) receptors [221]. By regulating the NR2B subunit of NMDA receptors, NaHS treatment improved the cognitive dysfunction associated with hepatic ischemia/reperfusion in hippocampus of rats [222]. Various studies demonstrated the protective effects of H_2_S on neurons against oxidative stress [223]. H_2_S is unlikely to scavenge oxidants by itself due to its low endogenous concentration [83], whereas it seems to exert potent antioxidant effects indirectly regulating GSH production through the upregulation of the Keap1/Nrf2/ARE pathway. In addition, in vitro and in vivo models demonstrated that activation of Nrf2 in astrocytes provides protection also in neurons, prompting the hypothesis that this effect is the primary factor leading to neuroprotection of both cortical and motor neurons [1]. Notably, both D-Cys and L-Cys, substrates for H_2_S synthesis showed to protect the neurons of the cerebellum from hydrogen peroxide (H_2_O_2_)-induced oxidative stress [81]. In human cultured neuron cells, H_2_S inhibits peroxynitrite, acting as an endogenous peroxynitrite scavenger [1]. Dysregulation of H_2_S metabolism has been described in different neurological diseases. Hu et al. showed reduced levels of H_2_S in striatum and substantia nigra in two different murine models of Parkinson’s disease [224]. NaHS treatment attenuated neuronal loss, lipid oxidation, accumulation of inflammatory markers, and improved movement dysfunction [224]. In addition, H_2_S sulfhydration plays a relevant role in Parkinson’s disease by activating the neuroprotective ubiquitin E3 ligase, parkin, responsible for clearance of toxic, misfolded proteins [82]. Parkin sulfhydration was found markedly depleted in the brains of patients with Parkinson’s disease, suggesting that this loss may be pathologic and hydrogen sulfide donors may be therapeutic [225]. Likewise, H_2_S levels were found reduced in the plasma of patients with Alzheimer disease (AD) compared to controls, and H_2_S levels correlated negatively with the severity of the disease [226]. Depletion of H_2_S and concomitant increase in homocysteine levels in AD have been attributed to a lack of S-adenosylmethionine (SAM), an allosteric activator of CBS. Moreover, NaHS treatments in murine models of AD reduced oxidative stress through Nrf-2 and provided protection against homocysteine-induced cognitive dysfunction [227]. In addition, recent studies have shown that inhalation of H_2_S protected against PD-induced movement dysfunction and prevented neuronal apoptosis and microglia activation in the nigrostriatal region. CBS overexpression has also been reported to protect against the 6-hydroxydopamine-induced model of PD [228]. On the other hand, H_2_S production by CBS has been found elevated in amyotrophic lateral sclerosis (ALS) both in mice tissues and cerebrospinal fluid of ALS patients. Similarly, high H_2_S levels have been observed in trisomy of chromosome 21 trisomy (Down syndrome), on which the CBS gene is located [82]. It is well documented in the literature that H_2_S exerts either antioxidant and anti-inflammatory effects or prooxidant and pro-inflammatory effects depending on its local concentration. Following a bell-shaped dose–response curve, H_2_S produces protective effects at a lower concentration and a variety of deleterious/cytotoxic effects at higher concentrations [83,91]. The varying effects of H_2_S reported in several lines of evidence could be due to the dual effects of H_2_S [82]. This has been widely suggested in investigations concerning various types of inflammatory processes, vasorelaxation/tension in aortic tissue, cell proliferation/apoptosis, as well as in the case of tumor promotion and inhibition. Since the concentration of H_2_S can vary considerably both within and between tissues and under differing conditions, it is likely that H_2_S could affect a complex array of biological responses that may challenge the capacity to make definitive biomedical and/or clinical interpretations or predictions. Li and Moore [229] closed their recent insightful review of the role of H_2_S in health and disease with a statement probing their articulated biological conundrum of how does one molecule display such widely differing effects and could it be due to different effects at differing concentrations. The answer to this seminal question is found within the framework of a hormetic dose–response interaction that is independent of biological model, tissue, and endpoint [1].

## 7. H_2_S Redox Signaling and Resilience

**Figure 3:**
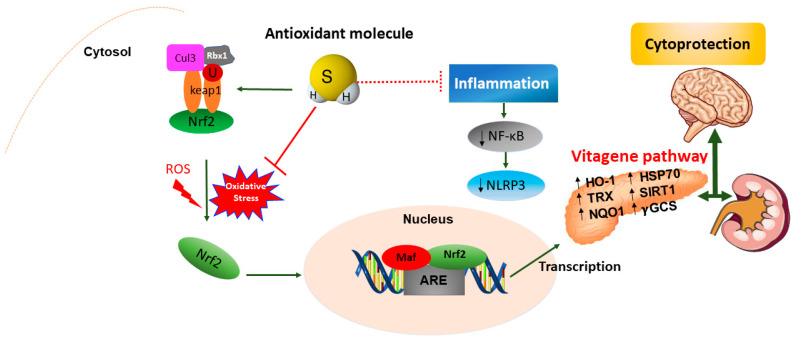
The modulation of the Nrf2-vitagene pathway by H_2_S. In physiological conditions, Nrf2 is bound to its inhibitor Keap1 and is restricted to the cytosol where it undergoes ubiquitination and proteasomal degradation via association with the Cul3-Rbx1-based E3/ubiquitin ligase complex. Under stress conditions, Nrf2 is released from Keap1 and is translocated into the nucleus where it binds to the phase 2 of ARE in heterodimeric combination with the Maf transcription factor in the DNA promoter region. The H_2_S antioxidant molecule blocks oxidative stress and NLRP3 inflammasome cascade by activating Nrf2 nuclear translocation and the transcription of cytoprotective (phase 2) vitagenes. The upregulation of the vitagene pathway such as HO-1, Hsp70, Trx, sirtuin Sirt1, NQO1, and γ-GCS improves brain health in neurological disorders. Nuclear factor-erythroid 2 p45-related factor 2 (Nrf2), Kelch-like ECH-associated protein 1 (Keap1), antioxidant response element (ARE), heme-oxygenase 1 (HO-1), heat shock protein 70 (Hsp70), thioredoxin (Trx), sirtuin 1 (Sirt1), NAD(P)H: quinone oxidoreductase 1 (NQO1), γ-glutamylcysteine synthetase (γ-GCS).

Emerging evidence has highlighted the crucial role of H_2_S in maintaining redox homeostasis, which occurs by modulating levels of cellular antioxidant enzymes and increasing the expression of the transcription factor nuclear erythroid-related factor 2 (Nrf2) during oxidative stress. The latter is an intracellular excess of reactive oxygen species (ROS) relative to depletion of antioxidant capacity of the cell [230]. Interestingly, Nrf2 is a master regulator of redox cellular stress response in various pathological states [1,231]. Under physiological conditions, Nrf2 is localized in the cytosol and regulated by its inhibitor Kelch-like ECH-associated protein 1 (Keap1). Recently, much evidence has demonstrated that H_2_S induces cytoprotection against oxidative stress by the stimulation of Nrf2, which accumulates and translocates into the nucleus where it binds to the antioxidant response element (ARE) inducing the transcription of multiple target genes, including phase II detoxification enzymes such as NAD(P)H: quinone oxidoreductase 1 (NQO1), heme oxygenase 1 (HO-1), thioredoxin, γ-glutamylcysteine synthetase, and glutathione S-transferase (GST) [232]. In line with this observation, Nrf2 encodes the vitagene antioxidant pathway which exists to counteract different forms of stress (e.g., oxidative, environmental, and mitochondrial stress). *Vitagenes* include heat shock protein 70 (Hsp70), heme oxygenase 1 (HO-1), γ-glutamylcysteine synthetase (γ-GCs), thioredoxin (Trx), and sirtuins (SIRTs) (Figure 3) [1,233,234,235] as biomarkers for stress adaptation, cross-tolerance, and resilience underlying hormesis or preconditioning [236] (Figure 3).

**Figure 4:**
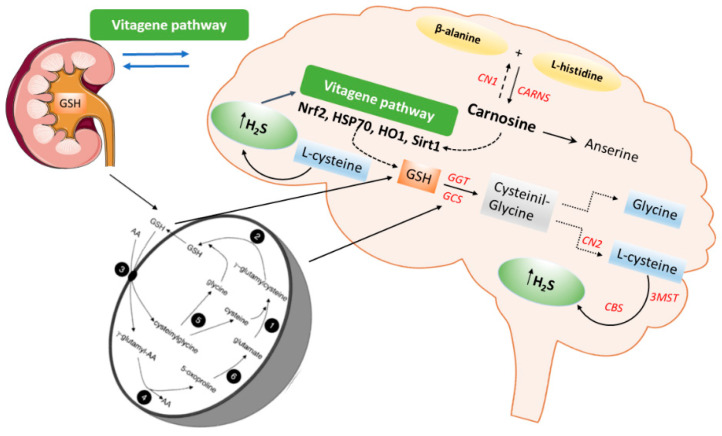
Schematic representation of the carnosine, glutathione, and hydrogen sulfide (H_2_S) pathways in the kidney-brain axis. γ-glutamyl transpeptidase (GGT) (3), γ-glutamyl cyclotransferase (4), dipeptidase (5), oxoprolinase (6), γ-glutamyl-cysteine synthase (1), and glutathione synthetase (2) operate in the Meister cycle to generate glutathione (GSH) and internalize amino acids (AA). GSH interacts with Cystathionine-β-synthase (CBS) and 3-mercaptopyruvate sulfurtransferase (3MST) to produce H_2_S.

For instance, the benefits of hormetic stimuli are well documented, such as preconditioning, dietary restriction as well as intermittent fasting in regulating endogenous H_2_S production by the conserved trans-sulfuration pathway (TSP) in a dose–response manner during ischemia reperfusion (I/R) injury [237,238,239]. Moreover, H_2_S can also act as a mimetic of dietary restriction and extend lifespan in yeast and worms [240]. Intriguingly, a growing number of studies have demonstrated that H_2_S decreases the ROS level in cardiomyocytes under ischemia/reperfusion injury in the setting of diabetes by relieving oxidative stress, and the ability of H_2_S to upregulate cellular antioxidants in the heart in a Nrf2-dependent manner [241,242]. Especially H_2_S-mediated cardioprotection increases in antioxidant (i.e., HO-1, Trx, Hsp70, and Hsp90) and anti-apoptotic (i.e., Bcl-2, Bcl-xL, and COX-2) signaling in a mouse model of pharmacological preconditioning [241]. These findings are consistent with the integrated view that cellular resilience is mediated by many distinct pathways that converge upon mitochondria to supply the extra energy for building resilience. Accordingly, several research groups have characterized the effect of H_2_S on mitochondrial activity and cellular bioenergetics [242,243,244]. These studies mentioned above demonstrated that H_2_S induces a U-shaped biological dose–response concentration typical of hormetic compounds in in vitro and in vivo models, with activation at lower concentrations and inhibition at higher concentrations [245]. Thus, the toxic and therapeutic effects of H_2_S depend on its endogenous concentration. Specifically, H_2_S is synthesized endogenously and mainly metabolized by a mitochondrial sulfide-oxidizing pathway including sulfide quinone oxidoreductase (SQR) and utilizes GSH as a thiophilic acceptor to produce GSSH [246]. Therefore, H_2_S produced in the mitochondria scavenges ROS [156]. In principle, H_2_S is a potent inhibitor of oxidative phosphorylation (OXPHOS) by its well-known ability to inhibit cytochrome *c* oxidase (COX) [247]. On the other hand, H_2_S therapy has been observed to preserve mitochondrial function in the heart muscle of rodents after I/R injury [238,248]. It has been well established that a low level of H_2_S administration increases the phosphorylation of protein serine/threonine kinase B (Akt) and enhances the nuclear localization of two transcription factors, nuclear respiratory factors-1 and -2, which are involved in increasing the levels of endogenous antioxidants, attenuating apoptosis, increasing mitochondrial biogenesis, and confer resilience against cellular stress [249]. Recently, the modulation of *vitagenes* elicited through H_2_S antioxidant molecule has taken on a considerable importance in preserving redox homeostasis, mitochondrial stress, protein quality control, and enhancing resilience in a hormetic dose–response manner during metabolic diseases such as diabetes and its complications. In this context, H_2_S provides antioxidant function by enhancing γ-glutamyl synthetase activity and upregulation of cysteine transport, restoring the levels of GSH in glutamate-mediated oxidative stress [148]. Some experimental studies suggested that administration of H_2_S attenuates high glucose-induced elevation in ROS production in renal mesangial cells and diabetic rat kidneys [250]. In addition, H_2_S treatment exerts protective effects in the kidney of type 1 diabetic rats, related to the suppression of oxidative stress through upregulation of superoxide dismutase (SOD) activity [251]. Interestingly, H_2_S mitigates diabetic renal damage by blocking mitochondrial Ca^2+^ permeability through the N-methyl-d-aspartate receptor-R1 (NMDA-R1) pathway [252]. Based on the concept of hormesis [253], in which low levels of H_2_S confer cytoprotective actions against oxidative stress, it can also cause toxicity when present in high concentrations [1,254]. Under oxidative stress, reactive sulfur species (RSS) are generated that can act as aggressive oxidizing agents. This phenomenon is mainly due to the high concentration of the H_2_S signaling molecule [255]. Thus, efforts have been made to identify suitable exogenous H_2_S donors. Indeed, some in vitro studies indicate that a high concentration of 0.1 and 1 mM NaHS induces pro-oxidant effects such as lipid peroxidation and protein carbonylation in human plasma [256]. In addition, in vivo studies demonstrate that NaHS improves diabetic state-induced muscle atrophy by increasing Akt/mTOR signaling and decreasing the expression of myostatin and the FoxO1/MuRF1/atrogin-dependent pathway [257]. Recent studies revealed a novel H_2_S-induced posttranslational modification, termed protein S-sulfhydration (also known as S-persulfidation) [87,258]. During this process, the –SH group of cysteine residues becomes covalently converted to a –SSH group, which can result in changes in the activity of the protein. This process importantly contributes to physiological and pathophysiological H_2_S-signaling. In this context, the S-sulfhydration reaction with H_2_S or hydrogen polysulfide (H_2_Sn) participation plays a crucial regulatory role in the biogenesis of RSS from endogenous and exogenous precursors and on regulatory properties of this reaction. On the other hand, these reversibly oxidized –SH groups are under the control of intracellular antioxidant pathways such as glutathione (GSH), cysteine (Cys), and thioredoxin (Trx) that actively participate to restore redox H_2_S homeostasis [259]. It is noteworthy that excessive levels of H_2_S can be countered by activation of the sulfide oxidation pathway, which involves the enzyme sulfide quinone oxidoreductase that oxidizes H_2_S and reduces coenzyme Q [260]. Several studies have reported that S-sulfhydration is one mechanism where H_2_S interacts directly with the Nrf2 pathway. In this respect, H_2_S has been shown to S-sulfhydrate Keap1 at the cysteine-151 residue, leading to Nrf2 dissociation and increased nuclear translocation and expression of antioxidant genes through binding to promoters’ ARE sites [232]. Furthermore, H_2_S can S-sulfhydrate Keap1 at the cysteine-226 and cysteine-613 residues, leading to Keap1 inactivation, Nrf2 release, and activation of Nrf2-dependent gene expression [261]. Additionally, NaHS upregulated Nrf2 nuclear translocation, and the transcription of the two key downstream antioxidant genes peroxiredoxin-1 and NAD(P)H dehydrogenase quinone 1 [262]. Most studies indicate that insufficient endogenous H_2_S concentration is implicated to increase oxidative stress leading to type 2 diabetes in mice and in humans [263,264]. Conversely, exogenous H_2_S promoted antioxidant effects by increasing Keap-1 and suppressing its ubiquitination, facilitating ubiquitin aggregate clearance via autophagy in the hearts of db/db mice [265]. In addition, recent studies demonstrated that H_2_S, in the form of a NaHS donor, may reduce high glucose-induced oxidative stress, inflammation, and apoptosis by activating the Nrf2/ARE pathway and may exert anti-apoptotic effects in diabetic myocardium by inhibiting c-Jun N-terminal kinase (JNK) and p38 MAPK pathways and activating PI3K/Akt signaling in vitro and in vivo [266]. Nevertheless, other recent studies showed that slow-releasing H_2_S donor, GYY4137, induces cardioprotection against myocardial ischemia and reperfusion injury by attenuating oxidative stress and apoptosis via activation of the PHLPP-1/Akt/Nrf2 pathway in mice [267], inhibition of the STAT3/HIF-1α signaling pathway [268], and activation of the AMPK (5′AMP-activated protein kinase)/mTOR signal pathway in high glucose-induced H9c2 cardiomyocyte damage [269]. Moreover, the GYY4137 donor exerts anti-inflammatory effects by suppression of the NF-kB and MAPK signaling pathway in Coxsackie virus B3-infected rat cardiomyocytes [270] as well as attenuating the development of diabetic cardiomyopathy via FoxO1 signaling pathway in vitro and in vivo [271]. Recent findings reported that the H_2_S–Nrf2–antioxidant proteins axis protects renal tubular epithelial cells and rescues cells from the native hibernator and from lipid peroxidation-mediated cell death under reoxygenation conditions [272]. Recently, Kimura et al. provided novel insights into generated potential redox regulators cysteine- and glutathione-persulfide species (Cys-SSH, GSSH) as well as signaling molecules such as H_2_S_2_ and H_2_S_3_ produced by 3MST in the presence of physiological concentrations of cysteine and glutathione, to maintain neuronal transmission, vascular tone, cytoprotection, inflammation, and oxygen-sensing [273]. In this scenario, it has been shown that H_2_S_2_ along with H_2_S_3_ shields neuronal cells from oxidative as well as carbonyl stress through exerting reduced synthesis of glutathione [101]. Additional studies suggested that H_2_Sn rather that H_2_S regulates the activity of the tumor suppressor phosphatase and tensin homolog (PTEN) and reduces blood pressure by dilating vascular smooth muscle [274] through the modulation of the Nrf2 pathway [275]. Interestingly, H_2_Sn activates transient receptor potential ankyrin 1 (TRPA1) channels by sulfhydrating two cysteine residues at the amino terminus of the channels [276] and the species also facilitates the translocation of Nrf2 to the nucleus to upregulate antioxidant genes by sulfhydrating its binding partner Keap1 to release Nrf2 [275]. It is well known that decreased endogenous H_2_S levels, redox imbalance, and oxidative damage are closely correlated with disease severity and progression in cardiac, neurological, pulmonary, gastric, nephrological, hepatic diseases, as well as in aging. Notably, Xie and coworkers detect significantly lower levels of plasma H_2_S in diabetic mice that is corrected by administration of exogenous H_2_S donor GYY4137 [277]. Intriguingly, GYY4137 exerts anti-atherogenic [277] and inflammatory effects [278] via the Nrf2/HO-1 pathway in mice. In addition, H_2_S induced NRF2 activity, the upregulation of antioxidant genes HO-1, Trx-1, and GSH, and the reduction in inflammation response by suppressing the NF-κB pathway [279] as well as apoptosis in rat models [280]. In these conditions of inflammation, activation of the cellular stress response represents an essential system that requires upregulation of the antioxidant vitagene pathway to preserve resilience and redox homeostasis of the cell in various pathological conditions [1,281,282,283]. Within this context, silent mating type information regulator 2 homolog 1 (SIRT1) is a NAD+-dependent deacetylase of lysine residue of the target protein. Mammals have seven different sirtuins, SIRT1–SIRT7 [284]. SIRT1 extends lifespan [285] and improves cell tolerance to inhibit environmental stress [286]. Recently, it has been shown that H_2_S is a novel SIRT1 activator by direct sulfhydration. Numerous experimental studies in vitro and in vivo indicate protective effects of H_2_S signaling through activation of Sirtuin’s family in different pathologies and, in particular, in type 2 diabetes and related complications. In this regard, NaHS increased SIRT1 and reversed biochemical, apoptotic, oxidant, and pathologic parameters characteristic of diabetic nephropathy, at a dose of 100 µmol/kg/day [287]. In vivo studies reported that hydrogen sulfide ameliorated reperfusion-induced oxidative stress and mitochondrial dysfunction via activation of Sirtuin3 signaling, thereby decreasing lung ischemia-reperfusion damage in rats with a model of type II diabetes [288]. Endogenous CSE/H_2_S directly sulfhydrated SIRT1, enhanced SIRT1 binding to zinc ion, then promoted its deacetylation activity, and increased SIRT1 stability, thus reducing atherosclerotic plaque formation [289]. In vitro studies suggested that H_2_S attenuates CSE-induced cellular senescence and apoptosis by improving mitochondrial function and reducing oxidative stress in alveolar epithelial cells [290] as well as protects against hyperglycemia-induced neuronal senescence and neurotoxicity in the mouse hippocampal cell line [291] via upregulation of the SIRT1 pathway. Compelling evidence reported that H_2_S plays a peculiar role in protecting the ageing kidney from antifibrosis and anti-apoptosis through the regulation of redox homeostasis. Consistent with this, SIRT1, which emerges as a major lifespan regulator, has been widely investigated in the cardiovascular system and nervous system, but it is rare in the urinary system. Recent studies have demonstrated that a low concentration of NaHS (25 μmol/L) could directly induce SIRT1 activation in a cell-free system, whereas chronic treatment of NaHS (50 μmol/kg/day) selectively improves the expression but not the activity of SIRT1 in the ageing kidney. SIRT1 also regulates lipid metabolism by modulating a great variety of signaling pathways such as PPAR-α, LXR, FXR, and SREBP signals [292]. It is noteworthy that SIRT3 is a crucial regulator of mitochondrial function. SIRT3 catalyzes the deacetylation of mitochondrial proteins, which in turn affects mitochondrial energy metabolism. SIRT3 is regulated by nutritional status and metabolic stress. In this context, a recent in vivo study has demonstrated that exogenous H_2_S supplement attenuated isoproterenol-(ISO-)-induced myocardial hypertrophy through SIRT3 protein in mice [293]. It is well observed that chronic NaHS treatment modulates Nrf2 downstream genes such as HO-1, SOD1, and SOD2, consequently enhancing resistance to oxidative stress in the ageing kidney. Chronic exogenous H_2_S treatment could protect the ageing kidney by reducing oxidative stress, decreasing collagen deposition, and enhancing Nrf2 nuclear translocation, as well as increasing endogenous H_2_S production. Reactive oxygen species are constantly generated during metabolic diseases such diabetes. Among various signals activated by ROS, the thioredoxin pathway is among the extensively investigated signaling cascades that mediate oxidative cell proliferation, inflammation, senescence, and survival [294,295]. Trx represent the primary defense mechanism against oxidative stress. It contains two redox-active cysteine residues that protect protein against unwarranted oxidant-mediated inter- or intra-molecular disulfide bond formation. Some studies have demonstrated that H_2_S exerts antioxidative effects on the cells through the regulation of the redox state of Trx and interference with the ASK1/P38 signaling pathway [296]. Importantly, H_2_S modulates cellular redox signaling via direct S-sulfhydration of the Nrf2-Trx pathway. Consistent with this notion, Trx is the major regulator enzyme of intracellular persulfidation that cleaves disulfides in proteins and acts as an S-denitrosylase [297]. Especially, cysteine-32 within Trx is responsible for the direct interaction of Trx and S-sulfhydrated proteins following the breakage of the hydropersulfide group [298]. Likewise, Trx can desulfhydrate the protein tyrosine phosphatases 1B (PTP1B), restoring endoplasmic reticulum (ER) stress response in an in vitro setting [299]. Several studies revealed that H_2_S preconditioning could protect mice against cerebral I/R injury by the induction of HSP70 and the PI3K/Akt/Nrf2 pathway [300]. The heme oxygenase 1 (HO-1), also referred to as Hsp32, is induced by various oxidative stimuli, including ROS, RNS, and RSS. Accordingly, HO-1 has been recognized also as dynamic sensors of cellular oxidative stress and modulators of redox homeostasis throughout the phylogenetic spectrum. In this light, H_2_S exerts a protective role by upregulating the antioxidant enzyme HO-1 expression in human kidney cells [301] (Figure 4).

In addition, recent in vivo studies reveal the nephroprotective effects of H_2_S on renal tissue through upregulation of antioxidant proteins and anti-inflammatory cytokines, as well as the expression of eNOS and iNOS via induction of the Nrf2/HO-1 pathway in renal injury [302] and in the spinal cord of rats [303]. Moreover, H_2_S could attenuate high glucose-induced myocardial injury in rat cardiomyocytes by suppressing the Wnt/β-catenin pathway and upregulating the expression of HO-1 and NQO1 [304]. In addition, H_2_S protected renal tissue against ischemia-reperfusion injury-induced lipid peroxidation, inflammation, and apoptosis, which may be attributed to the upregulation of HSP 70, HO-1, and HSP 27 [305]. H_2_S reduces myocardial fibrosis in diabetic rats, which is related to the inhibition of protein kinase Cα (PKCα), upregulation of HSP70 expression [306], and downregulation of the JAK/STAT signaling pathway [307]. Finally, H_2_S protects cells from oxidative stress [308] and delays programmed cell death by increasing the levels of antioxidant glutathione and HO1 expression [309]. Taken together, the data above convincingly indicate the crucial role of H_2_S as a potent antioxidant molecule that, at low concentrations, induces protective actions exploited through the redox modulation of the Nrf2 vitagene signaling pathway which may provide a novel potential therapeutic approach to confer resilience against oxidative stress, inflammation as well as apoptosis during pathological conditions such as diabetes and related complications.

## 8. Analytical Approaches to Quantify H_2_S in Biological Matrices

Hydrogen sulfide, present in mammalian tissues, plays a vital role in physiological and pathophysiological processes. The long identified toxic gas, which has also been confirmed as the third gaseous signaling molecule following NO and CO, plays important roles in various physiological and pathological processes. However, striking differences with orders of magnitude were observed for the detected hydrogen sulfide concentrations in biological matrices among different measurements in the literature, which lead to the uncertainty for examination of the biological relevance of hydrogen sulfide. The monitoring of the quality control of H_2_S donor drugs and the study on the pathophysiology and pharmacology mechanism of H_2_S on experimental animals and even on humans require for detection methods with high sensitivity, selectivity, precision, and accuracy. Various techniques for the analysis of low concentrations of H_2_S have been developed over the past 50 years. Some of the older methods are still used by health departments, but due to their very low sensitivity, they cannot be used in the quantification of very low concentrations of this gas in mammalian tissues useful for scientific research. Also, due to the variety of chemical species with multiple properties, make it difficult accurate and reliable measurements of hydrogen sulfide in biological matrix [310]. Among the various methods that have been established for the measurement of endogenous hydrogen sulfide, the most widely used are: colorimetry [311], gas chromatography [312], electrochemical measurements by electrodes selective for sulfide (ion-selective electrodes, ISEs) [313], polarographic sensors [314], estimation by fluorescent probes [315], and high performance liquid chromatography (HPLC coupled with ultraviolet, fluorescence or electrochemical detection) [316,317]. Currently, fluorescence derivatization is the most used method. Hydrogen sulfide has two possibilities to perform nucleophilic substitution on fluorescence probes to form a fluorescent derivative that can be detected using a fluorimeter coupled with high performance liquid chromatography (HPLC) [318,319]. For example, monobromobimane (MBB) is a common fluorescent reagent that reacts rapidly and completely with thiol groups [320,321]. However, these methods have obvious limitations, such as complex preparation processes, low specificity and sensitivity, and time-consuming procedures [310]. Another analytical technique considered most effective today for the analysis of substances at such low concentrations is MS/MS, the analysis with triple quadrupole mass spectrometry in particular. Due to its low molecular weight and simple chemical structure, hydrogen sulfide is not suitable for direct quantification by triple quadruple mass spectrometry and thus, chemical derivatization was used before detection by LC-MS/MS [322,323,324]. Compared to all other methods, HPLC-MS/MS methods can determine concentrations in the order of 10^−9^–10^−10^ molar, the derivatization reactions allow high selectivity, and with the use of deuterated standards, it can calculate the recovery very simply. Therefore, in particular as regards those studies aimed to define the signaling role of H_2_S, HPLC-MS/MS methods, based on derivatization probes, are the most promising methodology to address the need to detect very small amounts of H_2_S in mammalian tissues and in other biological matrices.

## 9. Conclusions

This evaluation has demonstrated that both H_2_S and carnosine can modulate oxidative stress and inflammation in the brain and the kidney, suppressing damage interactive synergies between these two organs. Substantial evidence has emerged that these two agents independently activate the Nrf2 transcription factor, which then leads to the formation of acquired resilience following the quantitative features of the hormetic dose response. These processes also may involve the occurrence of transcription factor crosstalk that is both dose- and biological context-sensitive. The process is a general one, and is proposed to account for observations that hormetic effects are independent of biological model, cell type, level of biological organization, inducing agent, and endpoint measured. The emerging findings of H_2_S and carnosine are consistent with similar mechanistic and dose response findings for a wide range of chemopreventive agents such as sulforaphane and EGCG that mediate their protective effects via Nrf2 activation within the context of the hormetic dose response.
